# Pulmonary Tuberculosis

**Published:** 1875-03

**Authors:** 


*Pulmonary Tuberculosis: Its Pathology, Nature, Symptoms, Diagnosis, Prognosis, Causes, Hygiene, and Medical Treatment.* By Addison P. Dutcher, M. D. Philadelphia: J. B. Lippincott & Co., 1875. Buffalo: Martin Taylor.

So much interest is attached to the subject of pulmonary tuberculosis that any contribution to the literature of the subject is eagerly scanned to see if anything new or important has been contributed to the subject. The reader who looks through Dr. Dutcher's work with this idea, will, we fear, be disappointed.

The author follows the generally accepted views with relation to the causation and pathology of the disease, and with but slight exceptions his treatment is that usually pursued by the profession. He places great reliance upon the red border of the gums, both a diagnostic and prognostic sign. Alcohol is entirely and severely condemned in treatment, it being considered either as an invention of the devil, or the prescription of a medical man who is himself addicted to its use.

As a general resume of the subject, the work possesses some value, and were many sentimental passages, which seem entirely out of place in a scientific work, left out, it would be of more value to the professional reader, who does not care to mix up sentiment and science, poetry and pathology.

